# Increasing the Contrast of the Brain MR FLAIR Images Using Fuzzy Membership Functions and Structural Similarity Indices in Order to Segment MS Lesions

**DOI:** 10.1371/journal.pone.0065469

**Published:** 2013-06-17

**Authors:** Ahmad Bijar, Rasoul Khayati, Antonio Peñalver Benavent

**Affiliations:** 1 Department of Biomedical Engineering, Shahed University, Tehran, Iran; 2 Center of Operations Research University Institute, Miguel Hernández University of Elche, Elche, Spain; University of Maryland, College Park, United States of America

## Abstract

Segmentation is an important step for the diagnosis of multiple sclerosis (MS). This paper presents a new approach to the fully automatic segmentation of MS lesions in Fluid Attenuated Inversion Recovery (FLAIR) Magnetic Resonance (MR) images. With the aim of increasing the contrast of the FLAIR MR images with respect to the MS lesions, the proposed method first estimates the fuzzy memberships of brain tissues (i.e., the cerebrospinal fluid (CSF), the normal-appearing brain tissue (NABT), and the lesion). The procedure for determining the fuzzy regions of their member functions is performed by maximizing fuzzy entropy through Genetic Algorithm. Research shows that the intersection points of the obtained membership functions are not accurate enough to segment brain tissues. Then, by extracting the structural similarity (SSIM) indices between the FLAIR MR image and its lesions membership image, a new contrast-enhanced image is created in which MS lesions have high contrast against other tissues. Finally, the new contrast-enhanced image is used to segment MS lesions. To evaluate the result of the proposed method, similarity criteria from all slices from 20 MS patients are calculated and compared with other methods, which include manual segmentation. The volume of segmented lesions is also computed and compared with Gold standard using the Intraclass Correlation Coefficient (ICC) and paired samples *t* test. Similarity index for the patients with small lesion load, moderate lesion load and large lesion load was 0.7261, 0.7745 and 0.8231, respectively. The average overall similarity index for all patients is 0.7649. The *t* test result indicates that there is no statistically significant difference between the automatic and manual segmentation. The validated results show that this approach is very promising.

## Introduction

Multiple sclerosis (MS) is a chronic neurological disease of the central nervous system (CNS), specifically involving the brain, spinal cord, and optic nerves. There are several strategies to determine if a person meets the long-established criteria for a diagnosis of MS and to rule out other possible causes of whatever symptoms the person is experiencing. These strategies include a careful medical history, a neurologic exam and various tests, including magnetic resonance imaging (MRI), evoked potentials (EP) and spinal fluid analysis [1].

MRI is the best imaging technology for detecting the presence of MS plaques or scarring (also called lesions) in different parts of the CNS [1]. MS lesions can appear as a hyperintense signal or as a hypointense signal depending on its properties and on the used MRI sequence. The potential MRI sequences for detection of white matter lesions are T1-weighted (T1-w), T2-weighted (T2-w), PD-weighted (PD-w), and FLAIR images. Multiple hyperintense lesions on T2-w and PD-w sequences are the characteristic MRI appearance of MS. A black hole (BH) is defined as any abnormal hypointensity as compared with normal-appearing white matter visible on T1-w sequences concordant with a region of high signal intensity on T2-w images. These so-called black holes have various pathological substrates depending, in part, on the lesion age. FLAIR MR sequences produce heavily T2-w images by nulling the signal from CSF. FLAIR images provide a better lesion contrast than do PD-w or T2-w images [2]. Previous researches have shown that the FLAIR sequence contains the most distinctive lesion-healthy tissue differentiation for the segmentation of white matter lesions.

The radiological criteria for MS include the number of lesions on the MRI, their locations and their sizes, and this quantitative information is also crucial for studying the progression of MS lesions and the effect of drug treatments. Consequently, segmentation of MS lesions from MR brain images is important for the diagnosis of the disease [3]. However, manual segmentation and analysis of these lesions from MR imaging examinations are usually time-consuming, error-prone, costly and greatly suffers from intra-observer and inter-observer variability [4], [5]. Errors occur due to poor hand-eye coordination, low tissue contrast and unclear tissue boundaries caused by partial volumes (where individual pixels contain more than one tissue type). Inconsistencies among qualified experts as to the extent of various structures are also common [6]. Therefore, there is a great interest to find new automatic and more effective methods or techniques to help clinicians in their decision-making regarding appropriate treatment of the disease. Automated procedures offer the advantage of producing consistent results in a much shorter operator time [7]. For this reason, automating the delineation of MS lesions from MR brain image is a complex and challenging task. Due to the existence of image imperfections such as noise, inhomogeneity effects and partial volume effects, segmenting MS lesions from MR brain images using only intensity values alone remains truly a difficult problem. Also, lesions cannot be simply modeled as normal anatomy, so they cannot be directly segmented using explicit anatomical templates. Consequently, segmentation of MS lesions is an extremely challenging task [8].

Over the last years, many automatic and semiautomatic approaches have been proposed for segmentation of the brain into different tissues, including MS lesions. These approaches include a variety of methods such as statistical, fuzzy, neural networks, fuzzy neural networks and so on. These methods are divided into supervised and unsupervised segmentation methods [9]. Supervised approaches are those based on using some kind of *a priori* information or knowledge to perform MS lesion segmentation. The supervised strategies group is further subdivided into two sub-groups: in the first group all approaches use atlas information [10]–[21], and therefore it is necessary to apply a registration process for the analyzed image to perform the segmentation; in the second group, all approaches perform an initial training step on features extracted from manually segmented images annotated by neuroradiologists [7], [22]–[32]. The methods in this second group employ the image intensities previously segmented by an expert, to train a classifier which segments the tissues and lesions of the MR images. Unsupervised strategies, where no *prior* knowledge is used, have two different subgroups: a sub-group of methods that segments brain tissue to help lesion segmentation [33]–[39]; and another sub-group that only uses the lesion properties for segmentation [40]–[44]. In the first sub-group, methods consist of either segmenting the tissue first and then the MS lesions, or segmenting the tissue and the lesions at the same time. In the second sub-group, the methods consist of directly segmenting the lesions according to their properties, without providing tissue segmentation. The advantage of segmenting the tissue is that neuroradiologists can also evaluate the GM tissue volumetry and monitor the progression of cerebral atrophy.

Four strategies are, therefore, proposed to deal with the automated MS lesion segmentation [9]: Supervised based on atlas, Supervised based on training, Unsupervised based on tissue and Unsupervised based on lesion. The inherent advantage of supervised algorithms is that they can automatically learn the characteristics of both normal tissue and lesions. However, their main problem is that they rely on having a good training set, which may be difficult to obtain. According to the procedure, two supervised strategies have been identified for introducing annotations into the algorithms: with or without using a registration step. The advantage of atlas-based approaches is that spatial information is inherently used, although registration is also a challenging task. On the other hand, training-based approaches allow for the use of the real features of tissues and lesions, but spatial information has to be introduced in a further step since it is not included in the training process. This group of unsupervised techniques has been subdivided into two different strategies according to the use of tissue information. The advantage of using tissue information is that it may help in localizing the lesions. However, the correct segmentation of the tissue is critical in these approaches. On the other hand, defining rules according to lesion features makes it possible to identify special lesions, although the rules may change according to the modality and scanning machine used [9].

In this paper, a new approach is proposed to increase the contrast of MR FLAIR images based on some image processing techniques for segmenting brain tissues from MS patients, without the need for any training set or any template. The brain image is considered in three parts, which include CSF, NABT and MS lesions, whose member functions of the fuzzy region are 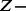
function, 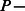
function and 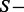
function, respectively. The fuzzy regions are found by Genetic Algorithm based on the maximum fuzzy entropy principle. The image can keep as much information as possible when it is transformed from the intensity domain to the fuzzy domain [45]. Consequently, the intersection points of the membership functions obtained are used to segment brain tissues. Research has shown that brain tissues are not segmented well when using the three-level threshold. So, each individual fuzzy region is used to classify brain tissues. In this way, MS lesions are determined by using a new Contrast-Enhanced image which is obtained from FLAIR image and its lesions membership image by means of SSIM indices. Also, CSF areas are segmented by applying a localized weighted filter to Dark membership image. The following sections explain: details of the research procedure including MR imaging type; manual segmentation of MS lesions; brain extraction; the use of the maximum fuzzy entropy; Genetic Algorithm; and SSIM indices to segment brain tissues. Finally, the proposed approach is introduced and evaluated using Gold standard. There are three significant advantages to the approach we propose: (1) our method is fully automatic so manual segmentation and training set are not required; (2) the proposed method increases the contrast of the MR-FLAIR images using some image processing steps in order to segment MS lesions;(3) only FLAIR image is used to segment MS lesions.

## Materials and Methods

### Patients and MR imaging

The proposed procedure in this research was implemented on MR images that were captured and used in [38]. This dataset contains 16 females and 4 males with an average age of 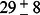, and was selected according to the revised Mc Donald criteria by Mc Donald 2005 [46]. Mean disease duration for the patients was five years. For all patients the same MR images were obtained via a Siemens 1.5T scanner. All images were acquired according to full field MRI criteria for MS [46] in T2-w, T1-w, Gadolinium enhanced T1-w and FLAIR in axial, sagittal and coronal surfaces. We selected the FLAIR images, especially the axial images, with lesions in deep, priventricular, subcortical, juxtacortical, and cortical areas. This selection was made because of greater lesion load and higher accuracy of FLAIR in revealing these MS lesions [47]. Although FLAIR is especially helpful for priventricular lesions closely apposed to an ependymal surface, where they may be obscured by the high CSF signal on T2-w images [47], Infratentorial lesions are better seen on PD-w images than on FLAIR [2]. Scan parameters for repetition time (TR)/echo time (TE)/inversion time (TI) and for FLAIR images were 9000/144/2500 ms. TR/TE for T1-w images were 424/10 ms and TR/TE for T2-w images were 3820/105 ms. Each image volume (patient data) consists of an average 40 slices with a 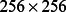 scan matrix. The pixel size is 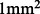, and the slice thickness is 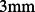 without any gap.

### Manual segmentation of MS lesions and brain extraction

The segmentation of MS lesions was performed manually by a neurologist and a radiologist in Flair images, with visual inspection of the corresponding T1-w and T2-w images. At first, manual segmentation was performed independently by two investigators, who were blinded to the study group. Then, a difference image of the two binary lesion maps was generated for each subject and together both experts together decided which differences were assigned to lesions or not. However, in some slices, the level of difference between the two binary lesion maps was unacceptable and the experts decided to segment these slices again. These manually segmented images were used as Gold standard [24] to evaluate the performance of the proposed method. Patients suffering from MS were divided into 3 groups according to the volume of their lesions [48]:

1. Patients with small lesion load (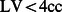)
2. Patients with moderate lesion load (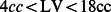)
3. Patients with large lesion load (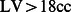)

To evaluate the proposed method, different types of images with different lesion volumes were applied. Also, brain extraction was performed using a fully automatic object-oriented approach [49]. This method was based on the regional-spatial characteristics of the brain in MR images. This algorithm consists of five steps. Firstly, the original image is converted to a binary image. Secondly, the morphological opening on the binary image is performed and tiny regions are eliminated. In the third step, three rectangular masks showing the cerebral regions are produced; the regions in the binary image which overlap with these rectangles are preserved and the rest are eliminated. In the fourth step, the final mask is generated by dilating selected regions and filling tiny holes. Finally, an image, which includes only cerebral tissues, is obtained by applying the resulting mask to the original image.

### Maximum fuzzy entropy based on probability partition

**Figure 1:**
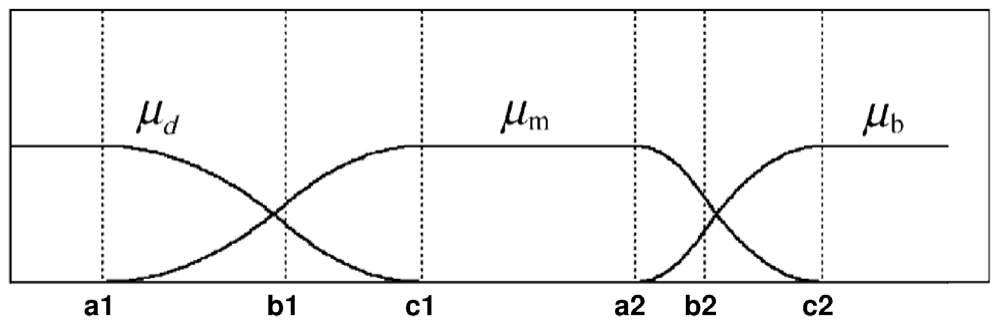
Membership function graph.

Let 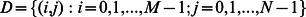 and 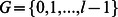, where 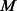, 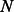 and 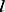 are three positive integers. Then a digitized image is considered a mapping 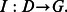 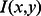 is the gray level value of the image at the pixel 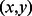.
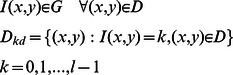
(1)

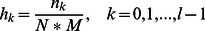
(2)where 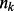 denotes the number of pixels in 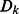. The following results can be formed
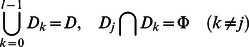


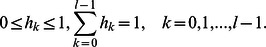


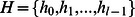
 is the histogram of the image, 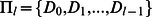 is a probability partition of 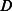 with a probabilistic distribution
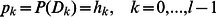
(3)The image has 256 gray levels 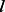 (in this paper). Here, three-level thresholding is used to segment the brain image: naming the two thresholds 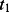 and 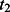; then, the image is segmented into three gray levels; and brain tissue segmentation is evaluated. In this gray level image, the domain 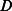 of the original image is classified into three parts: 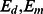 and 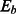. 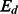 is composed of pixels with low gray levels; 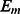 is composed of pixels with medium gray levels; and 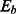 is composed of pixels with high gray levels. 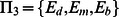 is an unknown probabilistic partition of 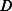, whose probability distribution is given below:
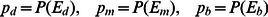
(4)A classical set is normally defined as a collection of elements that can either belong to a set or not. A fuzzy set is an extension of a classical set in which an element may partially belong to a set. Let A be a fuzzy set, where 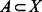 is defined as 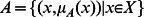 where 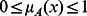 is called the membership function. The value of 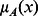 is the grade of x belonging to A. 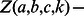
function, 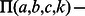
function and 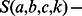
function are used to approximate the memberships of 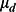, 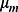 and 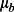 to the image with 256 gray levels. The membership functions have six parameters, namely 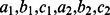. In other words, the two thresholds 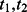, for three-level thresholding depend on 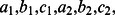 and the following conditions are satisfied:
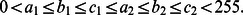
For each 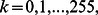 let
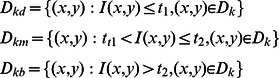
Then
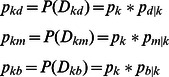
(5)When the pixel belongs to 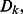 it is evident that 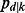, 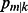, 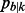 are the conditional probability of a pixel when it is classified into dark(d), medium(m) and bright(b) respectively, restricted to 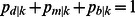;(k = 0,1,…,255).
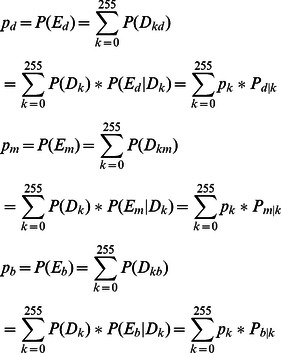
(6)In order to find the parameters 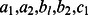 and 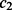 three membership functions are considered: 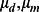 and 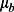, where 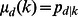, 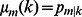 and 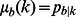. Obviously, 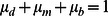, 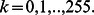 So, Eq. 6 is rewritten as
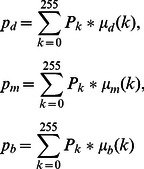
(7)The three membership functions are shown in Figure 1 [45].
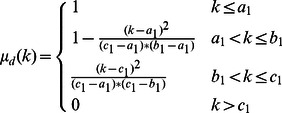
(8)

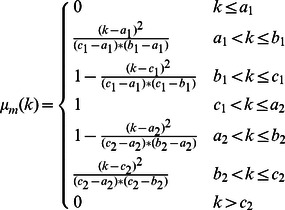
(9)

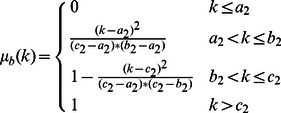
(10)where the six parameters 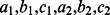 satisfy the following condition:
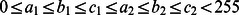
The fuzzy entropy function of each class is given below:
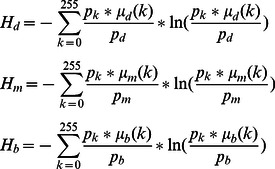
(11)Then, the total fuzzy entropy function is given as follows:
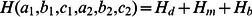
(12)We can find a combination of 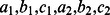 such that the total fuzzy entropy 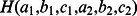 achieves the maximum value. In this paper, the procedure for finding the optimal combination of all the fuzzy parameters is implemented by genetic algorithms.

### Finding fuzzy parameters using Genetic Algorithm

The Genetic Algorithm (GA) is a stochastic global search method that mimics the metaphor of natural biological evolution [50]. A traditional, simple genetic algorithm has the following steps:

1. Create initial generation 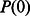, let 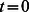.
2. For each individual 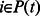, evaluate its fitness 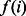.
3. Create generation 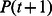 by reproduction, crossover, and mutation.
4. Let 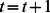. Unless 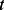 equals the maximum number of generations, return to Step 2

The user is required to specify the following parts for using GA: coding method, object function, the population size (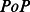), the cross-over probability 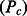, the mutation probability 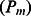 and the maximal number of generations (MaxGen). So we should present the encoding mechanism, the selection scheme, genetic operators, and the fitness function used. The first step is to encode the parameters 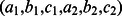 into an alphabet string. The parameters have to follow an increasing order: 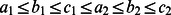. In our experiments, each image has 256 gray levels, i.e., the maximum value of 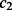 is 255. Therefore, the chromosome of the genetic algorithm in our experiment is encoded as six 8-bits strings that represent the value of all the parameters, respectively. If all the parameters 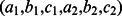 are generated randomly, it is possible that the parameters 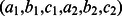 do not satisfy the criteria 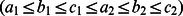. In such a case, we can assign zero to the value of object function for illegal chromosomes, which will not participate in the reproduction of next generation. The drawback of this method is that there are too many useless chromosomes in the searching space. Here we use a mathematical processing method to make all chromosomes legal. That is to say, every chromosome will satisfy the criteria 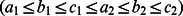. The method is described below:
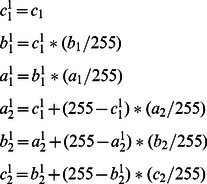
(13)Then the following condition is satisfied:
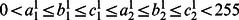
(14)We can find a combination of 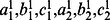 such that the total fuzzy entropy 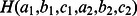 achieves the maximum value. Also, the parameters of the genetic algorithm are: Number of population: 300, Maximum number of generation: 0.5, 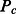
(probability of crossover) = 0.5, and 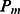
(probability of mutation) = 0.01.

### Structural Similarity Indices

We have used a structural similarity (SSIM) quality measure [51] from the perspective of image formation which is a function of luminance, contrast and structure. The algorithm's greatest appeal is that it matches human subjectivity. In particular, the SSIM Index, like the HVS (human visual system), is highly sensitive to degradations in the spatial structure of image luminances. The luminance of the surface of an object being observed is the product of illumination and reflectance, but the structures of the objects in the scene are independent of illumination. The structural information in an image is defined as those attributes that represent the structure of objects in the scene, independent of the average luminance and contrast. Since luminance and contrast can vary across a scene, local luminance and contrast are used.

Suppose 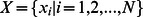 and 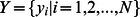 are two nonnegative image signals. Let 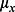, 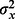 and 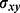 be the mean of X, the variance of X, and the covariance of X and Y, respectively. Approximately, 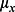 and 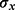 can be viewed as estimates of the luminance and contrast of X, and 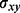 measures the tendency of X and Y to vary together, thus an indication of structural similarity. The general form of the SSIM index between signal x and y is defined as:
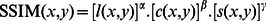
(15)where

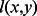
 is Luminance comparison measure. Luminosity is a comparison of the mean values of each image.
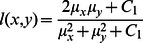
(16)The constant 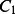 is included to avoid instability when 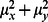 is very close to zero, and
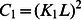
(17)where 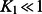 and the dynamic range of the elements of x and y is denoted by the variable 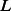.

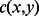
 is Construct comparison and is estimated as the standard deviation 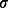. Structure comparison is done after local mean subtraction and local variance normalization.
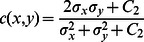
(18)The constant 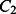 is included to avoid instability when 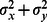 is very close to zero, and
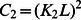
(19)where 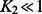 and the dynamic range of the elements of x and y is denoted by the variable 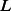.

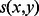
 is Structure comparison measure and is estimated from the image vector by removing the mean and normalizing it by standard deviation.
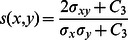
(20)where 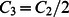.

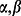
 and 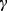 are used to adjust the relative importance of the three components..

This SSIM function, 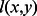, 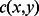 and 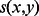 satisfy the following conditions:

1. Symmetry: 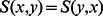;
2. Boundedness: 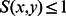;
3. Unique maximum: 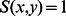 if and only if x
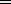
y (in discrete representations, 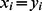 for all i = 1,2,…,N);

### Brain tissue segmentation

**Figure 2:**
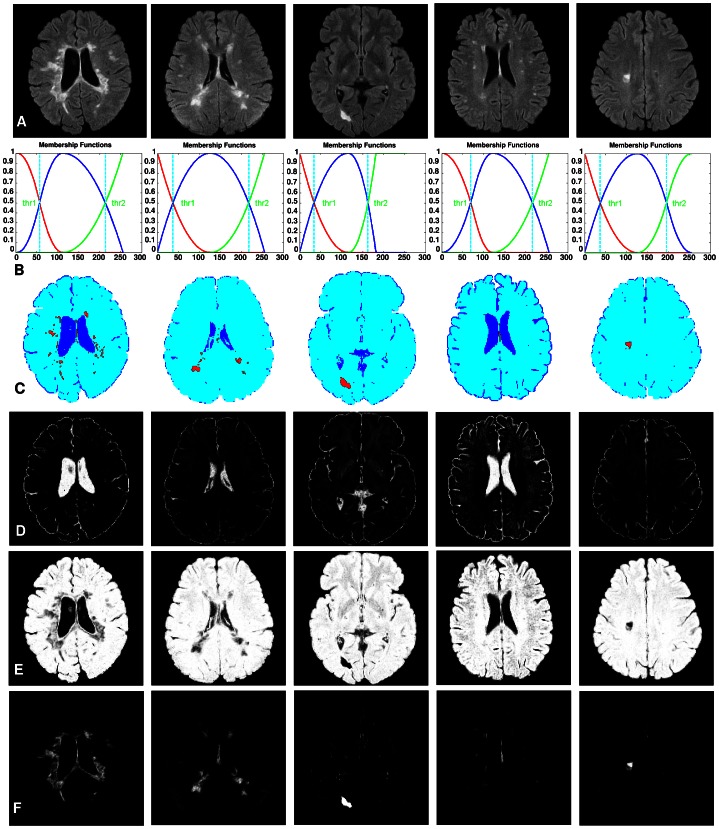
Brain tissue segmentation examples, using the three-level threshold. Each column shows the result of segmentation for a brain image with different lesion load. (a) Shows the original brain images. (b) The obtained member functions plots. (c) Shows the segmentation results using the three-level thresholding (maximum fuzzy entropy approach). (d) Dark membership images. (e) Medium membership images. (f) Bright membership images. (For interpretation of the references to color in this figure, the reader is referred to the web version of this article.)

**Figure 3:**
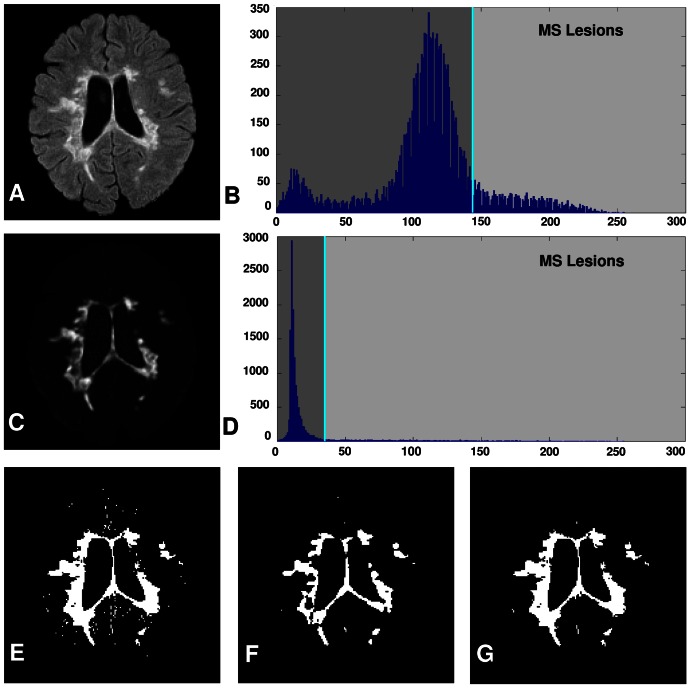
Contrast-Enhanced FLAIR image for segmentation of MS lesions. (a) Shows a typical brain image. (b) Histogram of original brain image. (c) Contrast-Enhanced image. (d) Histogram of Contrast-Enhanced image. (e) 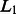: Lesion areas obtained from Contrast-Enhanced FLAIR image. (f) 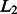: All candidate MS lesions obtained from bright membership image. (g) Result of MS lesions segmentation. (For interpretation of the references to color in this figure, the reader is referred to the web version of this article).

Fig. 2(a) shows five brain images with different lesion load. The membership functions obtained and the three-level threshold images are shown in Fig. 2(b) and (c), respectively. As seen in Fig. 2(c), brain tissues are not segmented well when using the three-level threshold. To make this clearer, the membership function values (dark, medium, bright) are computed for each pixel and shown in Fig. 2(d) to (f). In dark membership images, CSF areas have high intensity values in comparison with the rest of the brain areas. Also, in bright membership images, MS lesions have high intensity values in comparison with CSF and normal areas. These two membership images have been used to segment CSF and MS lesions. But, as seen in Fig. 2(f), there are a lot of pixels which do not belong to MS lesion class, but their bright membership values are similar to the MS lesions. Moreover, in manual segmentation, the possible lesions which are as small as one or two pixels in size are not usually considered as MS lesions by experts. These objects are recognized as noise and must be removed [38]. So, simple thresholding of the fuzzy membership images ends up with noise pixels in all classes. To tackle these problems, a new image created from the original brain image and its bright membership image are introduced. In this way SSIM indices [51] have been used to increase the contrast between lesions and the rest of the brain tissues (i.e., normal tissue and CSF) on FLAIR images as given below:
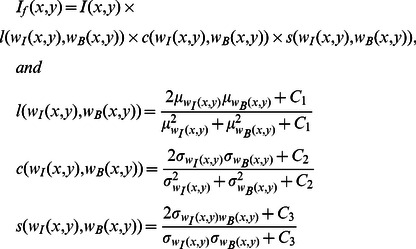
(21)where 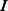, 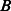 and 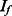 are respectively the original brain image, its bright membership image (with dynamic range of 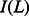) and the new created image; 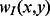 and 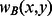 are two local windows (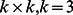) in image 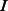 and 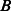, respectively, which are centered at pixel 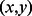. 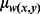 and 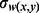 are mean and variance of the pixels which are placed in window 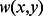. The constants 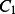 and 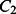 are included to avoid instability when 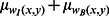 and 
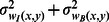
 are very close to zero and are equal to 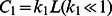 and 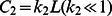, 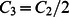. In other words, the constants 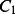, 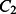 and 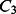 are small constants that aim to characterize the saturation effects at low luminance and contrast regions and ensure numerical stability when the denominators are close to zero. The obtained result for a typical brain image is shown in Fig. 3. As seen in Fig. 3, the contrast between MS lesions and the rest of brain tissues has been increased. A simple observation of the histogram of the Contrast-Enhanced image shows that it uniformly spreads across a large spectrum of values for MS lesions. Then, a primary mask (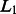) which determines MS lesions areas is created using *Adaptive thresholding* [52] from (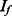). To have an accurate segmentation, *all* candidate MS lesions are determined by thresholding the bright membership image: 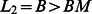. As mentioned before, brain tissues are not segmented well when using the three-level threshold, and as seen in Fig. 2e and f, most of the lesion pixels have been considered as other tissues; this means that they have a low bright membership function value. So, setting the 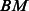 is very important for all types of brain images with different lesion load. If we lose a candidate MS lesion, which is an MS lesion, this means that it has been eliminated from the final segmentation result. So, to avoid any unwanted elimination of MS lesions, 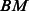 is set to 0.05, manually. So, in comparison with 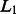, 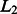 is a mask which contains 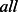 candidate MS lesions. Finally, To segment MS lesions, each individual area in 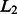 which overlaps with 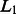 is selected as an MS lesion.

**Figure 4:**
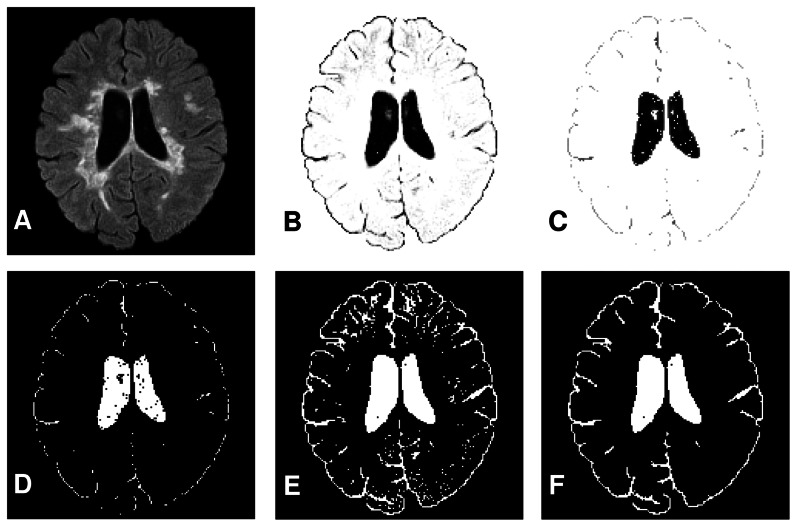
Segmentation of CSF areas. (a) Shows a typical brain image. (b) Dark membership image (to give more understanding, the obtained image has been inverted). (c) Result of applying the localized-weighted filter to dark membership image (the inverted result). (d) 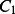: CSF areas obtained from filtered dark membership image. (e) 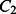: CSF areas obtained from dark membership image. (g) Result of CSF segmentation. (For interpretation of the references to color in this figure, the reader is referred to the web version of this article).

To segment CSF areas through the dark membership image, a localized weighted filter is used. At first, the dark membership image is filtered using
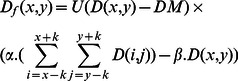
(22)Where 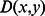 is the value of the dark membership image at pixel 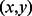, 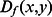 is its value in the filtered image, 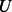 is the unit step function, 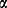 and 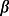 determine the effect of the pixel's neighborhood and the pixel itself, respectively, and 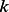 controls the neighborhood size. 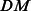 is considered as a threshold which determines candidate CSF pixels, and experimentally it is found that 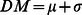 gives the best segmentation result, in which 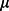, 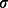 are the mean and standard deviation of non zero pixels of the 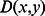, respectively. The filter parameters, i.e. 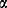, 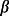 and 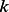 are the parameters used for adjusting the relative weights or contributions of neighborhood interaction, and have been experimentally set to 0.9, 0.6 and 3 for the best result. Non zero pixels of the 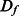 are then selected and considered as a primary mask of CSF areas (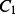). To accurately segment CSF areas, the dark membership image is also thresholded: 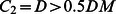. Finally, to segment CSF areas, each individual area in 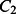 which overlaps with 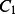 is selected as CSF areas. The result obtained for a typical brain image is shown in Fig. 4.

### Algorithm

**Figure 5:**
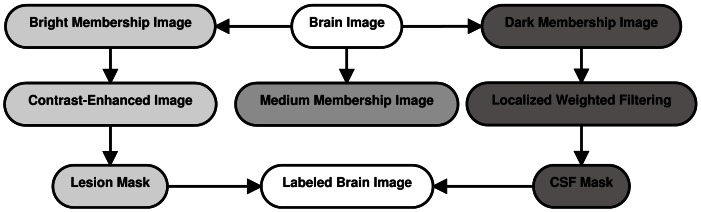
Block diagram of the proposed approach for fully automatic segmentation of MS lesions.

Based on the explanations given above, the block diagram of our method for MS lesion segmentation is shown in Fig. 5 and summarized below:

1. Segmentation of brain image.
2. Approximation of brain image memberships (dark, medium, and bright) by maximizing fuzzy entropy (the procedure to find the optimal combination of all the fuzzy parameters is implemented by genetic algorithm).
3. Segmentation of brain tissues
  - Segmentation of MS lesions:
    - Detection of MS lesion areas by thresholding the Contrast-Enhanced FLAIR image (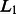).
    - Selection of *all* candidate MS lesions by thresholding the bright membership image (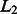).
    - Selection of areas which are located in the 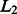 and overlap with 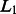 as MS lesions.
  - Segmentation of CSF regions:
    - Detection of CSF areas by thresholding the filtered dark membership image (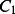).
    - Selection of candidate CSF areas by thresholding the dark membership image (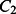).
    - Selection of regions which are located in the 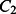 and overlap with 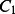 as CSF regions.
  - After segmentation of MS lesions and CSF areas, other pixels are labeled as normal tissues.

### Evaluation

**Figure 6:**
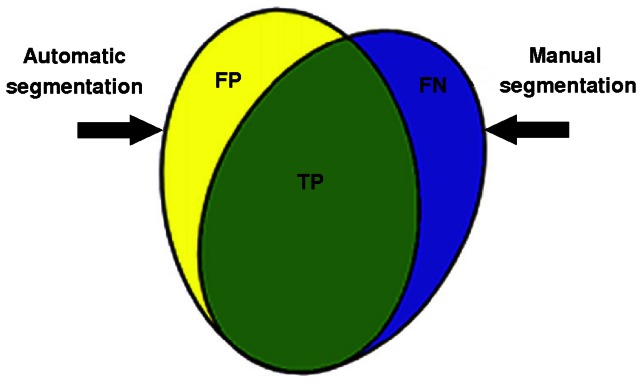
TP, TF, FP, and TN values are shown based on comparison between segmented regions by proposed method (Automatic segmentation) and manual segmentation.

Results of lesion segmentation based on the proposed method are compared with the Gold standard. To evaluate the proposed method, similarity criteria (SI) [53], overlap fraction (OF) and extra fraction (EF) [54] criteria are considered and computed for all 20 patients. SI is a criterion for the correctly segmented region relating to the total segmented region in both the manual segmentation and the image segmented using the proposed method. The OF and the EF specify the areas that have been correctly and falsely classified as MS lesions areas with respect to the MS lesion areas in manual segmentation. Similarity index, overlap fraction, and extra fraction are obtained, respectively, by Eq.(23) (see Fig. 6):
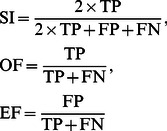
(23)where: TP stands for true positive voxels, FP for false positive voxels, and FN for false negative voxels. For a good segmentation, SI and OF should be close to 1 and EF should be close to 0. A value of more than 0.7 for SI practically represents a very good segmentation in this field [55]. Also, the mean values of the similarity criteria are categorized to the three patient categories and then, volumetric comparison of lesion volume between fully automated segmentation and Gold standard are performed using Intraclass Correlation Coefficient (ICC). Moreover, the paired samples *t* test is used to evaluate the consistency between computerized and manual segmentation. The original hypothesis is that there is no significant difference between the two groups of lesion areas segmented by different methods.

## Results

**Figure 7:**
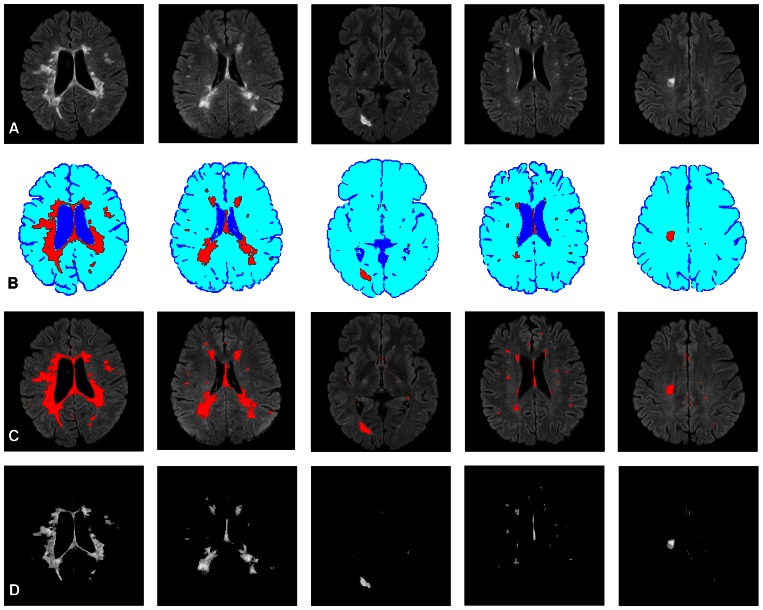
Brain tissue segmentation examples. **Each column shows the result of the proposed method for a brain image with different lesion load.** (a) Original brain images. (b) Rresult of automatic segmentation. (c) Results of the automatic MS lesion segmentation overlaid on brain image. (d) Extracted lesions. (For interpretation of the references to color in this figure, the reader is referred to the web version of this article).

The proposed algorithm was implemented on different FLAIR images using a PC with 2.5 GHz Pentium 4 processor and 512 MB RAM. The results of the proposed method for five slices with different lesion loads are shown in Fig. 7. Segmented brains from typical original FLAIR images and results of the proposed method for brain tissue segmentation are shown in Fig. 7 (*a*) and Fig. 7 (*b*), respectively. It is apparent that there is a good correlation between the input images (Fig. 7 (*a*)) and the resulting image (Fig. 7 (*b*)), indicating the acceptable performance of the suggested algorithm in detecting the lesion borders as well as the CSF regions. The results for the automatic MS lesion segmentation overlaid on brain images are shown in Fig. 7 (*c*). Extracted lesions are also shown in Fig. 7 (*d*).

The evaluation of the results was performed qualitatively and quantitatively as follows: the quality performance of the results was confirmed by the neurologist and the radiologist separately; then, in the quantitative evaluation step, the similarity criteria (i.e., SI, OF, and EF) were calculated for all slices. Mean values of the lesion volumes (LV) and similarity criteria are given in Table 1 for each patient data and for all images in the data set (last line of Table 1). As seen in this table, patient no. 1 has the lowest values for both OF and EF. Although it is expected to achieve a low OF value for patients with small lesion load, achieving low values for both OF and EF indicates a high number of False Negative pixels in this case. Also, mean values of the similarity criteria for each patient group are given in Table 2. As seen in this table, SI, OF and EF are improved with an increase in lesion load. The results of volumetric comparison of lesions between the proposed method and the Gold standard are also presented in Table 2. This table shows that according to the value of ICC, the accuracy of the proposed method is increased for patients with large lesion load. The differences between the computerized method and manual segmentation are analyzed using the paired samples *t* test, with the level of significance set at 5%. The paired *t* test between the areas extracted by the two methods achieves a P value of 0.131. The result indicates that the areas worked out by the two methods are highly correlated without a significant difference at the averages.

**Table 1:** Mean values of lesion volumes (LV), similarity criteria and mean value of segmentation time (T) for each patient data and for all images in data set (last line of the table) obtained using the proposed method.

Patient No.	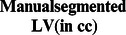	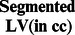	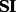	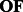	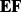	T(Sec.)
1	0.873	0.699	0.7252	0.6529	0.1478	24.81
2	1.611	1.797	0.7165	0.7579	0.3575	27.25
3	1.884	2.112	0.7132	0.7564	0.3646	28.29
4	2.547	2.868	0.7224	0.7680	0.3581	25.42
5	2.991	2.619	0.7476	0.7011	0.1745	26.73
6	3.054	3.360	0.7325	0.7692	0.3310	26.19
7	3.888	3.723	0.7253	0.7099	0.2477	27.68
8	6.438	6.069	0.7633	0.7414	0.2013	28.17
9	9.057	9.726	0.7692	0.7976	0.2763	25.78
10	9.855	10.266	0.7726	0.7890	0.2527	27.24
11	10.359	11.613	0.7739	0.8207	0.3003	29.77
12	11.283	12.102	0.7808	0.8091	0.2635	28.11
13	13.803	12.648	0.7810	0.7483	0.1680	25.56
14	15.414	16.170	0.7823	0.8015	0.2476	28.31
15	16.173	17.676	0.7769	0.8130	0.2799	25.56
16	17.232	16.029	0.7739	0.7469	0.1833	26.12
17	17.907	18.819	0.7713	0.7909	0.2600	28.62
18	21.189	22.047	0.8190	0.8356	0.2049	26.35
19	26.331	25.890	0.8240	0.8171	0.1661	27.51
20	28.587	29.421	0.8262	0.8383	0.1909	28.94
Mean	11.0238	11.2827	0.7649	0.7732	0.2488	27.18

**Table 2:** Similarity criteria and volumetric comparison of lesions for each patient group.

Patient category	N	Similarity criteria			Correlation analysis		
		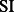	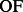	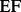	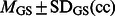	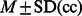	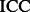
Small lesion load	7	0.7261	0.7308	0.2830	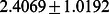	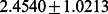	0.963
Moderate lesion load	10	0.7745	0.7858	0.2433	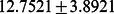	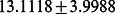	0.971
Large lesion load	3	0.8231	0.8303	0.1873	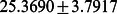	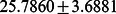	0.980

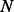
: number of patients in each group, 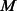: mean, 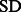: standard deviation,

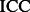
: Intraclass Correlation Coefficient (two-way mixed model with absolute agreement definition and 95% confidence interval).

## Discussion

**Figure 8:**
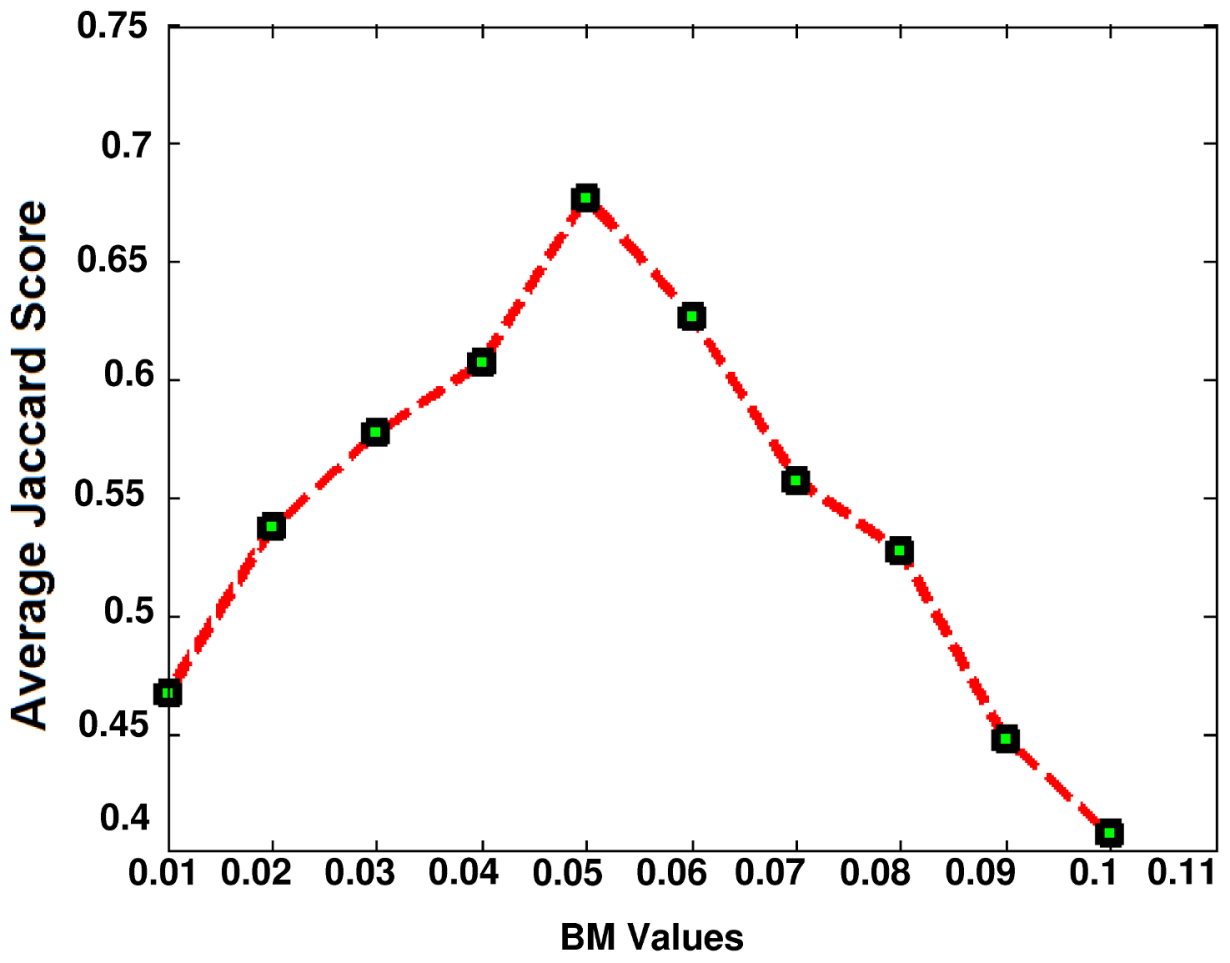
The average of the Jaccard Scores for different values of the BM parameter (in the interval [0.01, 0.1]).

In this paper, a new approach for fully automatic segmentation of brain tissues in MR FLAIR images of MS patients is proposed. At first, the brain image is partitioned into three parts, including dark (CSF), gray (normal tissues) and white part (MS lesions), whose member functions of the fuzzy region are 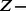
function, 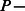
function and 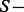
function, respectively. Membership functions (MF) can either be chosen by the user arbitrarily based on the user's experience (MF chosen by two users could be different depending upon their experiences, perspectives, etc.), or be designed using machine learning methods (e.g., artificial neural networks, genetic algorithms, etc.). For this application, modeling requires continuously differentiable curves and therefore smooth transitions, which the trapezoids do not have. The fuzzy regions are determined by using maximizing fuzzy entropy. The procedure to find the optimal combination of all the fuzzy parameters is implemented by genetic algorithm, which can overcome the computational complexity problem. The intersection points of the obtained membership functions are considered to segment three parts. Research has shown that brain tissues are not segmented well when using the three-level threshold. So, each individual fuzzy region is used to classify brain tissues. It should be noted that the value of the BM parameter is very important for the segmentation of MS lesions; to find the optimal value, an empirical approach is used to maximize the Jaccard scores of the abnormality detection results for a set of values in the interval [0.01, 0.1]. As can be seen in Fig. 8, the optimal value obtained is 0.05. Although using a lower BM value increases the number of True Positive pixels and decreases the number of False Negative pixels, it causes an increase in the number of False Positive pixels. Also, using a greater value for BM parameter decreases the number of True Positive and False Positive pixels, and increases the number of False Negative pixels.

Manual segmentation is used to evaluate the proposed method via similarity criteria (i.e., SI, OF, and EF) in a data set of MR FLAIR images of 20 MS patients. Other researchers who have used similar evaluation methods of evaluation (i.e., SI) are shown in Table 3. It should be noted that these researchers have used real data set and manual segmentation for the evaluation of their methods. As seen in Table 3, the MS lesion segmentation algorithms use different MR images to segment MS lesions and are evaluated with different databases. So, direct comparison between our proposed method and those reported here through the value of SI, without considering Images and Databases is not justified. Methods reported in Table 3 will be reviewed below.

**Table 3:** Similarity index (SI) values for the proposed method and the other methods.

#	Article	Method	Similarity index (SI)	Images	Database
1	Datta *et al.* [27]	Supervised	0.75	PD, T1, T2, FLAIR	14v
2	Admiraal-Behloul *et al.* [48]	Supervised	0.75	PD, T2, FLAIR	100v
3	Bijar *et al.* [39]	Unsupervised	0.75	FLAIR	20 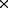 12 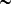 20 s
4	Khayati *et al.* [38]	Unsupervised	0.7504	FLAIR	20 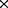 12 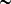 20 s
5	Datta *et al.* [43]	Supervised	0.76	PD, T1, T2, FLAIR	22v
6	Proposed Method	Unsupervised	0.7649	FLAIR	20v
7	Sajja *et al.* [26]	Supervised	0.78	PD, T2, FLAIR	23v
8	Anbeek *et al.* [24]	Supervised	0.8	PD, T1, T2, FLAIR, IR	20 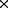 38 s
9	Anbeek *et al.* [25]	Supervised	0.808	PD, T1, T2, FLAIR, IR	10 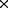 5 s

s: slices, v: volume.

The reader is referred to the [9], [58] for complete explanations about methods reported here.

Admiraal-Behloul *et al.* [48] suggested a fully automatic segmentation method for quantifying white matter hyperintensity in a large clinical trial on elderly patients. The algorithm they introduced combined information from three different MR images including PD, T2-w and FLAIR and FCM algorithm for the clustering process. The approach demonstrated very high volumetric and spatial agreement with expert delineation. To initialize and guide the FCM algorithm, they used brain templates, where prior distributions of the tissue types were supposed to be known. The success of a template-based segmentation algorithm depends on the outcome of the template. Bijar *et al.* [39] presented an automatic segmentation of MS lesions in FLAIR MR images. The proposed method estimated a gaussian mixture model with three kernels as CSF, normal tissue and MS lesions. To estimate this model, an automatic Entropy-based EM algorithm was used to find the best estimated model. Then, Markov random field (MRF) model [56] and EM algorithm were used to obtain and upgrade the class conditional probability density function and the apriori probability of each class. After the estimation of Model parameters and apriori probability, brain tissues were classified using bayesian classification. Khayati *et al.* [38] combined an adaptive mixture method (AMM) [57], MRF and a Bayesian classifier to simultaneously classify the three main brain tissues and the MS lesions using only FLAIR images. In particular, they first segmented the brain into four classes: WM, GM, CSF and ‘others’. Afterwards, inside the ‘others’ class, lesions were dealt with as outliers which were not correctly explained by the model. Sajja *et al.* [26] used PD-w, T2-w, and FLAIR MR images to segment MS lesions, which involved techniques such as Parzen window classifier, morphological operations, hidden Markov random field-expectation maximization (HMRF-EM) algorithm, and fuzzy connectivity. A similar approach was employed by Datta *et al.* [43] to identify black holes in MS. The method proposed by Anbeek *et al.* [24] was a supervised pixel classification which used different information, including voxel intensity and the spatial information, to classify voxels by a K-nearest neighbor (KNN) classifier. This technique assigned a probability to each voxel for being part of white matter lesion. The SI was then used to determine an optimal threshold on the probability map to segment the images. Their approach showed high accuracy compared to other methods for a similar task.

Compared to the above-mentioned methods our proposed algorithm does not need any training set or template. There is further information about those methods which classified their input database into different lesion load in Table 4. Anbeek *et al.* [24] and Admiraal-Behloul *et al.* [48] made use of FLAIR images for the segmentation of white matter lesions in patients of (Mean 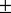 SD: 65.6
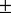
7.7) years old. In comparison, we used FLAIR images for the segmentation of MS lesions in younger patients (Mean 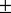 SD: 29
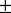
8), which were also used by Khayati *et al.* [38] and Bijar *et al.* [39]. For the patients with small lesion load, Anbeek *et al.* [24], Admiraal-Behloul *et al.* [48], Khayati *et al.* [38] and Bijar *et al.* [39] reached values of 0.5, 0.7, 0.7253 and 0.7262 for SI, respectively, while we obtained a value of 0.7261 for SI, according to Table 4. As seen in Table 4, our proposed method improves the value of SI by about 2% for the patients with a moderate lesion load in comparison with others. Also, there was no improvement in SI of patients with large lesion load compared with supervised methods. However, on average, an increase of about 1.49% in the SI value for all patients was seen in our proposed approach, compared to Admiraal-Behloul *et al.* [48], Khayati *et al.* [38] and Bijar *et al.* [39] and no improvement achieved compared to Anbeek *et al.* [24], which is a supervised method. Furthermore, lesions that are smaller than six voxels were excluded by Admiraal-Behloul *et al.* [48], while we do not exclude any lesions. If we ignore the lesions that are smaller than six voxels, the results of fully automated segmentation will be improved, because lesions which are possibly as small as one or two pixels in size are not usually considered as MS lesions by experts in manual segmentation.

**Table 4:** SI values for the proposed method and the other methods.

Method	Patient category			
	Small lesion load	Moderate lesion load	Large lesion load	All patients
Anbeek *et al.* [24]	0.50	0.75	0.85	0.8
Admiraal-Behloul *et al.* [48]	0.70	0.75	0.82	0.75
khayati *et al.* [38]	0.7253	0.7520	0.8096	0.7504
Bijar *et al.* [39]	0.7262	0.7531	0.8101	0.75
Proposed method	0.7261	0.7745	0.8231	0.7649

Finally, our findings about lesion load in FLAIR images, mentioned in Table 2, are consistent with previous studies by Anbeek *et al.* [24], Admiraal-Behloul *et al.* [48], Khayati *et al.* [38] and Bijar *et al.* [39]. They suggested that better SI and CC were associated with bigger T2-w lesion load.

Also, intraclass correlation test revealed a strong correlation between the proposed method and manual segmentation (ICC = 0.996). Statistical analysis using the *t* test for paired samples showed that there was no significant difference between the obtained results and manual segmentation.

**Figure 9:**
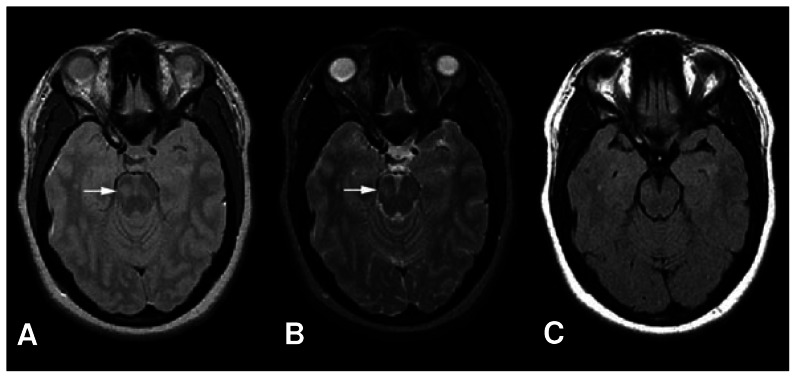
A typical Infratentorial lesion. (a) PD-w, (b) T2-w, (c) and FLAIR images of a patient with remitting relapsing multiple sclerosis (RRMS) demonstrate a pontine lesion (arrows) that is not demonstrated on the FLAIR sequence [2].

Because of partial volume effect, the edges of the tissues or lesions are not well defined and consequently their correct delineation is not easy. It becomes even more difficult when the operator delineates small or irregular lesions and, as a result, correction of the partial volume effect is necessary [41]. The most prominent partial volume effect can be seen at the interface of lateral ventricles, especially in T2-w and PD images, and also in subarachnoid CSF spaces in T1-w enhanced images. Since we made use of FLAIR images and theoretically in FLAIR images, CSF signals are suppressed in these regions, we are able to ignore the partial volume artifact in our study. However, we expect to use some corrective measures, such as morphological operators, connectivity principles and the integration of explicit anatomical models of ventricles, which are useful and reduce this artifact [59]. It is reminded that FLAIR images are less sensitive in the depiction of lesions involving brainstem and cerebellum, so lesion load may be underestimated in the posterior fossa [60] (see Fig. 9).

There was no significant field inhomogeneity in the data set, so, we did not use any field inhomogeneity correction method as the pre-processing step. We repeated the experiments using a bias field correction method and there was no considerable improvement in the results. Although the probable reason may be the use of the SSIM index in enhancing the contrast of the FLAIR image locally and then the detection of lesion areas, we cannot claim that the affect of global intensity inhomogeneity has been canceled by the proposed method.

As the proposed algorithm requires no training and is based on estimating three fuzzy membership functions for all classes (i.e., CSF, NABT, and lesions) through the Genetic algorithm, which is a stochastic global search method, this type of method is less dependent on image intensity standardization and can be used with different scanners.

As future research, we intend to use different fuzzy membership functions for MS lesion segmentation and hope it gives better and more accurate results. Also, a simple observation of the Contrast-Enhanced image's histogram shows that it is uniformly spread across a large spectrum of values for MS lesions, which could be used to detect MS lesions subtypes in future studies.
